# Bispecific Antibodies for IFN-β Delivery to ErbB2^+^ Tumors

**DOI:** 10.3390/biom11121915

**Published:** 2021-12-20

**Authors:** Vladislav S. Rybchenko, Anna A. Panina, Teimur K. Aliev, Olga N. Solopova, Dmitry S. Balabashin, Valery N. Novoseletsky, Dmitry A. Dolgikh, Petr G. Sveshnikov, Mikhail P. Kirpichnikov

**Affiliations:** 1 Department of Bioengineering, Shemyakin & Ovchinnikov Institute of Bioorganic Chemistry, Russian Academy of Sciences, 117997 Moscow, Russia; dbalabashin@mail.ru (D.S.B.); dolgikh@nmr.ru (D.A.D.); kirpichnikov@inbox.ru (M.P.K.); 2 Department of Chemistry, M.V. Lomonosov Moscow State University, 119234 Moscow, Russia; 3 Blokhin National Medical Research Center of Oncology, Ministry of Health of the Russian Federation, 115478 Moscow, Russia; solopova@msn.com; 4 Russian Research Center for Molecular Diagnostics and Therapy, 117638 Moscow, Russia; petrsveshnikov40@gmail.com; 5 Department of Biology, M.V. Lomonosov Moscow State University, 119234 Moscow, Russia; valery.novoseletsky@yandex.ru

**Keywords:** ErbB2, IFN-β, bispecific antibody

## Abstract

The main aim of our work was to create a full-length bispecific antibody (BsAb) as a vehicle for the targeted delivery of interferon-beta (IFN-β) to ErbB2^+^ tumor cells in the form of non-covalent complex of BsAb and IFN-β. Such a construct is a CrossMab-type BsAb, consisting of an ErbB2-recognizing trastuzumab moiety, a part of chimeric antibody to IFN-β, and human IgG1 Fc domain carrying knob-into-hole amino acid substitutions necessary for the proper assembly of bispecific molecules. The IFN-β- recognizing arm of BsAb not only forms a complex with the cytokine but neutralizes its activity, thus providing a mechanism to avoid the side effects of the systemic action of IFN-β by blocking IFN-β Interaction with cell receptors in the process of cytokine delivery to tumor sites. Enzyme sandwich immunoassay confirmed the ability of BsAb to bind to human IFN-β comparable to that of the parental chimeric mAb. The BsAb binds to the recombinant ErbB2 receptor, as well as to lysates of ErbB2^+^ tumor cell lines. The inhibition of the antiproliferative effect of IFN-β by BsAb (IC50 = 49,3 µg/mL) was demonstrated on the HT29 cell line. It can be proposed that the BsAb obtained can serve as a component of the immunocytokine complex for the delivery of IFN-β to ErbB2-associated tumor cells.

## 1. Introduction

The development and research of new therapies for tumors with the overexpression of the ErbB2 receptor are urgent tasks. Recently, research on the use of type I interferons in the treatment of oncological diseases has intensified. Cytokine therapy is aimed at activating the cells of the immune system to fight against tumors, but it has drawbacks that limit its use, which manifest themselves in a number of side effects. Currently, a number of studies are being carried out on the use of IFN-β in oncology aimed at reducing the systemic effect of this cytokine. Among all cancer types, breast cancer is the most common, accounting for 13.1% of the total incidence. As a cause of death, it ranks in5th place, taking 522,000 lives annually, which is 6.4% of total breast cancer cases [1]. Depending on the presence of oncological markers on the malignant cell surface, three type of breast cancer can be distinguished: ER/PR^+^, ErbB2^+^ and triple negative. The therapeutic strategies for each of those types differ; in particular, the ER/PR^+^ type is treated with hormone therapy [2]; the triple negative form lacking all three markers can be cured with the use of immune checkpoint inhibitors [3]. ErbB2^+^ tumors are presented in 11–30% of total breast cancer cases; their treatment involves the use of ErbB2-targeted therapeutics [4].

ErbB2 is a protein from the EGFR family that plays an essential role in cell growth, proliferation and migration [5]. However, ErbB2 overexpression is associated with oncological processes and poor prognosis. Moreover, an increase of the ErbB2 expression level has also been seen in bladder, pancreas, ovarian, colon, kidney, esophagus, prostate and other types of cancer [6]. The probability of relapse for ErbB2^+^ breast cancer in I–II stages is 2.7 times higher than for ErbB2 negative types, and the chance of metastasis development is 5.3 times higher [7]. An induced increase in the *ErbB*2 transcription level in mice results in the appearance of epithelial-origin tumors and their growth [8]. Besides that, some somatic mutations of the *ErbB*2 gene, termed activating mutations, increasing ErbB2 phosphotyrosine kinase activity or dimerization effectiveness are found in ErbB2^+^ breast cancer patients [9]. The inhibition of phosphatase activities, crucial for ErbB2 signal activity suppression, may also drive malignant cell transformation [10]. Taking all of these facts in account, both the increase of ErbB2 level expression or ErbB2 malfunctioning due to mutagenesis or other mechanisms are important factors in oncogenesis. Nowadays, ErbB2-targeted therapy is one of the most effective in cancer treatment. The breakthrough occurred after the registration of humanized monoclonal antibody trastuzumab (Tz) (trademark Herceptin). This antibody binds to the fourth extracellular domain and disrupts receptor dimerization, preventing the activation of the PI3K-AKT and RAS-MAPK pathways, which are important for cell proliferation and growth [11]. The other two mechanisms of trastuzumab action are antibody-dependent cellular cytotoxicity and complement system activation [12]. The combination of trastuzumab and chemotherapy is considered a gold standard for ErbB2^+^ breast cancer treatment [13]. Besides trastuzumab, there are other targeted molecules for ErbB2, for example, pertuzumab, a humanized monoclonal antibody against the second extracellular domain of ErbB2. As well as trastuzumab, pertuzumab disrupts ErbB2 dimerization and involves the immune system to fight tumor [14]. Despite the effectiveness of the use of anti-ErbB2 antibodies, in some cases tumors become resistant to the therapy [15]. That is why the research and development of new therapeutics for ErbB2^+^ cancer treatment is an important task for scientists around the world.

Interferons are cytokines with antiproliferative, antiviral, proapoptotic and antiangiogenic activities. Though the effectiveness of antiproliferative and proapoptotic effects depends much on the cell line used for experiments, it is known that interferon-beta (IFN-β) suppresses growth and cell proliferation and induces apoptosis with higher effect than other interferons. It has been observed at least for glioma [16], hepatocarcinoma [17] and breast cancer [18] cell lines. One of the effects described for IFN-β is cell cycle arrest in the S-phase [19]. IFN-β also increases the level of the expression of proapoptotic proteins Fas, FasL and TRAIL, which are important for apoptosis activation [20]. Local effects of IFN-β includes tumor infiltration by macrophages [21], the enhancement of antigen presentation by increasing MHC I expression level [22], the activation of NK-cells [23], the generation of NO by macrophages [24] and angiogenesis suppression [25]. One of the aims of cytokine therapy is the activation of immune system cells in order to improve tumor recognition and the mediation of cytotoxic reactions. Nevertheless, the practical use of cytokines is limited due to side effects, whose severity depends on the type of a cytokine and its dosage. The use of viral vectors carrying the *IFN-β* gene may reduce the systemic side effects of IFN-β during administration [21]. The construction of immunocytokines is another way to avoid nonspecific reactions and deliver cytokine to the tumor site. In particular, the effectiveness of an immunocytokine, which consists of anti-ErbB2 antibody trastuzumab and IFN-β, was already demonstrated by a group of researchers [26].

Bispecific antibodies (BsAbs) are engineered to recognize two different antigens at the same time. Due to that property, they are used to crosslink immune system cells, mostly T-killers, to tumor cells and activate them [27,28]. However, BsAbs could also be considered a vehicle for cytokine delivery for cancer treatment. In the present work, we describe the construction of a molecular complex of IFN-β and bispecific antibody (BsAb) anti-ErbB2 and anti-IFN-β for the targeted therapy of ErbB2^+^ solid tumors. To implement this concept, one of the components of the BsAb should bind and neutralize the activity of IFN-β, and the other component specific to ErbB2 will redirect the complex to the tumor site. We suppose that this approach will allow the achievement of high local concentrations of IFN-β at the tumor site and metastasis and to avoid the severe side effects associated with high doses of interferon administration. The main aim of the present work was to construct BsAb based on previously obtained monoclonal antibodies to ErbB2 and to IFN-β with neutralizing activities and to study its properties.

## 2. Materials and Methods

### 2.1. Analysis of a Panel of Hybridomas Producing Antibodies to IFN-β. Sequencing Genes of Antibodies Neutralizing IFN-β

A panel of 21 hybridomas producing antibodies to IFN-β was obtained according to the standard protocol. IFN-β-specific antibodies to HuIFN-β expressed in *E. coli* cells (E. coli-HuIFN-β), as well as to IFN-β expressed in CHO cells (CHO-HuIFN-β, PeproTech, UK), were found. Testing was carried out by ELISA as follows: antigens (E. coli-HuIFN-β and CHO-HuIFN-β) were adsorbed onto the solid phase overnight at 4 °C in PBS, pH 7.2, at a concentration of 2 μg/mL and 50 μL/well. After blocking with BSA and washing, they were incubated with polyclonal antibodies against mouse immunoglobulins conjugated with horseradish peroxidase (Impact, Russia). After incubation at 37 °C and washing, a TMB solution was added to the wells, which were incubated for 20 min at RT. The reaction was stopped by the addition of 4.8% H_2_SO_4_. The presence of a specific immune response was assessed by the optical density at 450 nm (OD450) using a Multiscan multichannel spectrophotometer. Indirect ELISA using commercial typing sera also determined the sub-isotypes of B1–B21 antibodies: goat antibodies against mouse immunoglobulins of different isotypes. The determination of the Kd of antibodies against IFN-β was carried out according to the method of Friguet [29] and others using the Klotz equation [30]

Ao/(Ao − A) = 1 + Kd ∗ 1/a(1)

where Ao is the optical density measured for antibodies in the absence of antigen; A—optical density for free antibodies in a mixture with steady thermodynamic equilibrium, and a—the concentration of IFN-β.

The activity of antibodies was determined in tests allowing the assessment of the inhibition of the antiproliferative action of IFN-β. MCF7 cells were seeded at a cell density of 5000 cells/100 μL of suspension per well; dilutions were prepared: IFN-β −1000 U/mL (giving about 50% inhibition of cell growth), IFN-β −1000 U/mL with monoclonal Ab from 50 μg/mL (molar ratio 1 >> 100), and monoclonal Ab 50 μg/mL in complete growth medium DMEM with 2% fetal calf serum. Each dilution was done in three repeats. As positive controls, wells without IFN-β: 0 (medium) and 0 + 50 (medium with a maximum antibody concentration of 50 μg/mL) were used. Negative controls were IFN-β wells without antibodies (IFN-β 1000 U/mL). Mixtures of IFN-β with antibodies were incubated in a CO_2_ incubator at 37 °C to form immune complexes. The prepared solutions were added to the wells of the plates, incubated for 72 h, then MTT was added to a final concentration of 0.05%, and the plates were placed in a CO_2_ incubator for 4 h at 37 °C. After incubation with MTT and washing, DMSO was added and incubation continued at room temperature for 15 min. The optical densities in the wells were determined using a Thermo Multiscan EX spectrophotometer at a wavelength of 560 nm.

### 2.2. Obtaining, Sequencing and Validation of Genes of Antibodies Neutralizing IFN-β

To obtain variable domains of mAbs, total RNA was isolated from cells (10^7^) of a corresponding hybridoma line with TRIzol (Gibco BRL, Waltham, MA, USA) according to the manual, and reverse-transcription was performed with the reverse transcriptase MMuLV and oligo (dT_18_) primer. The amplification of genes of the variable domains of the heavy and light chains (VH and VL) of mAbs encoded by the obtained cDNA was performed by PCR with a set of primers complementary to the regions of constant domains of mAbs (Table S1). PCR fragments were cloned into the pAL2-T vector (Evrogen, Moscow, Russia) and sequenced (Evrogen, Moscow, Russia). At least 10 VH and VL clones were examined. Nucleotide sequence analysis was performed by Chromas, CLC Sequence Viewer, and Gene Runner software. To confirm the correctness of the determination of the primary structure of the variable domains of mAbs, the method of peptide mass fingerprint was used. After separation by electrophoresis according to Laemmli in 12% polyacrylamide gel of L- and H-chains, tryptic and chymotryptic hydrolysis of proteins was carried out. Mass-spectrometric analysis of enzymatic hydrolysates of proteins was performed on an Ultraflex TOF/TOF instrument (Bruker Daltonics, Billerica, MA, USA). Correlation of mass spectra with amino acid sequences of proteins was performed using the BioTools 3.1 software (Bruker Daltonics, Billerica, MA, USA).

### 2.3. Construction of DNA Sequences Encoding Chimeric L- and H-Chains of Antibody B16

DNA fragments encoding VL and VH of B16 mAb were combined with the Cκ domain of the light chain and the CH1 domain of the human IgG1, respectively, by SOE-PCR with gene-specific primers. Simultaneously, a Kozak sequence, sequences for leader peptides and restriction sites *Nhe*I and *Xho*I for the cloning of chimeric antibody fragments into an expression vector pcDNA3.4 were introduced. Primers for SOE-PCR are presented in Table S2. The ligation of the variable domains of the light chains with the kappa domain was performed using SOE-PCR. The joining of the variable and constant domains of the H chain was carried out by introducing the recognition site of the restriction endonuclease *Bsp*120I at the 3′-end of the VH sequence and at the 5′-end of the CH1 domain of the H chain of human IgG1. The full-size L and H chimeric B16 nucleotide sequences were cloned into the expression vector pcDNA3.4.

### 2.4. Obtaining and 3D Modeling of Humanized B16 Antibody

The humanization of the B16 antibody was performed using the CDR-grafting method [31]. The IMGT database was used for the search for potential donors of FR-regions. IGHV3-23*04 and IGKV1-9*01 human germline Ig genes were found to share 80% and 65% identical amino acid sequences with murine B16 variable domains. After identifying CDRs and FRs according to Kabat numbering, murine FRs were replaced by the IGHV3-23*04 and IGKV1-9*01 corresponding regions. In order to prove that the replacement of the FRs of murine B16 did not affect variable domain structure and CDR-loop positioning significantly, Rosseta homology modeling and molecular dynamics simulation were performed.

Homology modeling with the aid of the web-service Rosetta Antibody (https://rosie.graylab.jhu.edu/antibody accessed on 19 December 2021) [32] was used to obtain the structures of variable domains. Further optimization was performed with a molecular dynamics (MD) simulation. This procedure allowed the optimization of the hydrogen bonds and salt bridges between protein residues and a solvent. Simulations were performed with the GROMACS software [33] (charmm36 force field, T = 300 K, 0,15 M NaCl, 11 ns). The inspection of MD trajectories showed that models reached equilibrium conformation during the first nanosecond. The next 10 ns of trajectories were considered with cluster analysis to detect the most representative conformations for every model. Trajectory visualization was performed with the VMD software [34]. Structure comparison was performed with the Maestro software (12.7, Schrodinger LLC, New York, NY, USA).

### 2.5. Construction of Humanized hB16 Neutralizing anti-IFN-β Antibody

The DNA sequences of the humanized variable domains of the hB16 antibody were synthesized by a chemo-enzymatic method (Evrogen, Moscow, Russia). Fragments that encode light-chain variable domain and heavy-chain variable domain sequences of humanized hB16 antibody were generated by PCR, separated from other reaction products by DNA-electrophoresis and purified on CleanUp standard kit (Evrogen, Russia) columns. The nucleotide sequences of VL and VH were joined with Ck and CH1-CH3 nucleotide sequences of human IgG1 by SOE-PCR, digested with *Nhe*I and *Xho*I endonuclease restriction enzymes and cloned into a pcDNA3.4 vector.

### 2.6. Construction of Vectors for Bispecific Antibody Production

Fragments encoding the variable domains of the Tz antibody against ErbB2-receptor were synthesized by a chemo-enzymatic method from pairwise overlapping oligonucleotides TzHF1—TzHR7 and TzLF1—TzLR6 (Tables S3 and S4). The purified fragments were cloned into the pAL2-T vector (Evrogen, Russia) and sequenced. VH and VL for Tz antibody were amplified by PCR with oligonucleotides TzHF1, TzHR7 (VH) and TzLF1, TzLR6 (VL). DNA fragments encoding F10 antibody leader peptides containing a restriction site *Nhe*I were amplified with primers F10LidLR (L chain), F10LidHR (H chain) and CMVF. Fragments encoding the leader peptide, variable and constant domains were obtained by SOE-PCR. Full-length L and H chains with leader peptides were cloned into the pcDNA3.4 expression vector. Knob-into-hole mutations [35] were introduced into anti-ErbB2 and B16 heavy chain genes by site-directed mutagenesis. For fragment amplification, pcDNA3.4-Tz and pcDNA3.4-B16 plasmids were used as templates, HTR1, HTF1, HTR2, HTF2, KTR1, KTF1, StTyR1 and StTyF1 primers (Table S5) and Tersus-polymerase (Evrogen, Moscow, Russia) were used; annealing temperature was set at 60 °C, and the number of cycles was 15. The obtained DNA fragments were cleaved by *Nhe*I and *Xho*I enzymes, purified by DNA electrophoresis, extracted from 1% agarose gel using CleanUp standard DNA extraction kit (Evrogen, Moscow, Russia) and cloned into a pcDNA3.4 plasmid vector prepared in the same manner. The plasmids containing knob-into-hole mutations were used for further heavy-chain crossover introduction. For B16 and anti-ErbB2 crossover-VL recombination, VH and kappa domain sequences were amplified with primers CMVF, B16LcdcR for B16VH, CMVF, TzHVdcR for TzVH and UniKapdcF and pcDNAR for kappa-domain, respectively (Table S6). After purification they were joined by SOE-PCR (T_m_ = 55 °C) and purified again by electrophoresis. Light-chain genes with crossover were cloned in the pcDNA3.4 vector and sequenced. In order to obtain a crossover-H chain gene, the VL of B16 and anti-ErbB2 antibodies were amplified with primers B16LcdcR, CMVF for B16VL and TzLVdcR and CMVF for TzVL, respectively (Table S6). Fragments were digested by *Nhe*I and *Bsp*120I and cloned into previously prepared pcDNA3.4 vectors, carrying B16 and Tz heavy-chain genes with knob-into-holes mutations (pcDNA3.4-TzHcr and pcDNA3.4-B16Hcr) digested with the same enzymes. Plasmids pcDNA3.4-TzLcr and pcDNA3.4-B16Lcr with light-chain crossovers and pcDNA3.4-TzL and pcDNA3.4-chimB16L were cleaved by *BamH*I and *Xho*I and used for the ligation of the IRES-element flanked by *BamH*I and *Nhe*I sites and heavy-chain genes flanked by *Nhe*I and *Xho*I sites.

### 2.7. Expression and Purification of Recombinant Antibodies

For the production of antibodies, a transient expression was performed in CHO cells using a pair of bicistronic vectors containing the genes of the L and H chains of the Tz antibody and the B16 antibody. A total of 24 h before transfection, CHO cells were subcultured at a concentration of 4 × 10^6^ cells/mL into CD OptiCHO medium (Invitrogen, Waltham, MA, USA), 8 mM L-glutamine (Invitrogen, USA) and 0.1% pluronic F68 (Gibco, USA). For transfection, 18 μg of DNA and 15 μL of FreeStyle MAX transfectant (Invitrogen, USA) were used. Cultivation was continued for 10–14 days at 37 °C and 8% CO_2_ with constant stirring on an ELMI S3.20L orbital shaker (ELMI, Riga, Latvia) at 130 rpm until the number of living cells in the culture decreased to 0.3 × 10^6^ cells/mL.The culture liquid was centrifuged, dialyzed against PBS and concentrated for purification on a 5 mL MabSelect SuRe LX Protein A Resin chromatographic column (GE Healthcare, Chicago, IL, USA). Isolation was performed according to the manual.

### 2.8. SEC Analysis

Size exclusion chromatography (SEC) of the purified proteins was conducted on a Superdex 200 10/300 GL column (GE Healthcare) at a flow rate of 0.4 mL/min in 100 mM Tris-HCl at pH 8.0 and 150 mM NaCl.

### 2.9. Validation of Recombinant Antibodies by Indirect ELISA

Indirect ELISA was used to determine the ability of the chimeric antibodies to interact with IFN-β in glycosylated and non-glycosylated forms compared to the prototype mouse antibody. After the adsorption of antigens onto the solid phase in PBS (0.5 μg/mL), wells were blocked with 5% BSA in PBS, washed; the studied antibodies were added, incubated at RT for 1 h and washed with 0.05% Tween 20 in PBS. A conjugate of monoclonal antibody 4G7 against human Ig light-chain kappa domain (Bialexa, Moscow, Russia) with horseradish peroxidase at a dilution of 1:75,000 was added, incubated at RT for 1 h and washed with 0.05% Tween 20 in PBS, and tetramethylbenzidine was added. The reaction was stopped by adding 10% sulfuric acid. Optical absorption was measured at 450 nm.

### 2.10. Indirect ELISA with Cell Lysates

SKOV3-ErbB2 (ATCC^®^ HTB-77), HT29 (ATCC^®^ HTB-38) cells, after washing with cold PBS, were lysed with RIPA buffer supplemented with protease inhibitors 1 mM PMSF and 1 mM aprotinin. RIPA buffer composition: 20 mM Tris-HCl (pH 7.5), 150 mM NaCl, 1 mM Na_2_EDTA, 1 mM EGTA, 1% NP-40, 1% sodium deoxycholate, 2.5 mM sodium pyrophosphate, 1 mM β-glycerophosphate, 1 mM Na_3_VO_4_, 1 μg/mL leupeptin. The precipitate was sonicated for the most complete extraction of membrane proteins and centrifuged for 10 min at 12,000 rpm and 4 °C. ELISA was performed as described above, with some variations. Cell lysates were sorbed onto a plate at a concentration of 10 μg/mL in PBS, 50 μL per well. BsAb were titrated from 800 ng/mL with a dilution factor of 2 in PBS-AT (0.01 M KH_2_PO_4_, 0.1 M NaCl, 0.2% BSA, 0.1% Tween 20). Trastuzumab/Herceptin antibodies (10 μg/mL) were used as a positive control.

### 2.11. “Sandwich” ELISA

“Sandwich” ELISA was used to determine the concurrent binding of BsAb to IFN-β and ErbB2 receptor. IFN-β (1 μg/mL) was sorbed onto the solid phase in an amount of 100 μL/well and incubated with BsAb (1 μg/mL) in serial dilutions. After washing, biotinylated recombinant ErbB2 (200 ng/mL) and avidin-horseradish peroxidase conjugate (150 ng/mL) were added. A monospecific antibody against the ErbB2 receptor (Herceptin), a recombinant antibody against the ErbB2 receptor (trastuzumab), and a full-length antibody B16 against IFN-β were used as controls.

### 2.12. Determination of the Neutralizing Activity of Antibodies

For the analysis of recombinant Ab, we used human tumor cells of the intestinal adenocarcinoma line HT29, which do not express ErbB2, and human peripheral blood mononuclear cells. Serial dilutions of BsAb, chimeric and humanized B16-based antibodies were prepared, as well as murine B16 antibody, which neutralizes IFN-β, as a positive control. Recombinant glycosylated IFN-β (Pharmapark, Moscow, Russia), 3 ng/mL, was added to the antibodies. The cultivation was carried out for 5 days. The number of living cells was assessed using the MTT test [36]. Each measurement was carried out in 4 repeats, and the average value was found. The neutralizing activity of antibodies was expressed as a percentage of the rate of cell proliferation without IFN-β and was calculated by the formula:

% neutralization = (Ai − A_0_)/(A_100_ − A_0_) × 100% (2)

A_i_ is the average optical density in the wells with ith concentration of the antibody; A_0_ is the average optical density in the wells with IFN-β without antibodies; A_100_ is the mean optical density in the wells without IFN-β and antibodies.

## 3. Results

### 3.1. Analisis of Hybridomas Producing Antibodies to IFN-β

**Figure 1:**
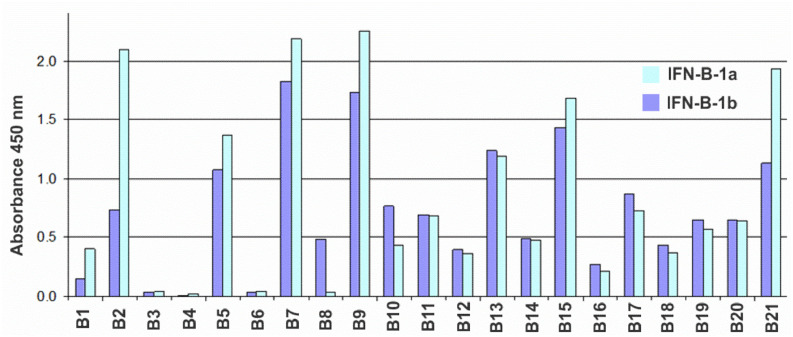
Histogram of the reactivity of antibodies B1–B21 against IFN-β according to the indirect ELISA with sorbed CHO-HuIFN-β-1a (blue bars) and E. coli-HuIFN-β-1b (dark blue bars) at an antibody concentration of 30 ng/mL.

A panel of hybridomas producing murine monoclonal antibodies against recombinant human IFN-β expressed in *E. coli* cells was prepared according to conventional techniques. Antibodies were tested by ELISA. To detect cross-reactions, E. coli-HuIFN-β, CHO-HuIFN-β, E. coli-HuIFN-α and E. coli-HuIFN-γ were used as antigens for binding to the antibodies under study. As a result of the analysis, it was found that none of the obtained antibodies cross-reacted with either IFN-α or IFN-γ. Testing of antibodies by indirect ELISA for binding to glycosylated (expressed in CHO) and non-glycosylated (expressed in *E. coli*) forms of IFN-β showed that all antibodies with the exception of B8 recognize non-glycosylated and glycosylated IFN-β forms with comparable binding levels (Figure 1).

The sub-isotypes of B1–B21 antibodies were also determined by indirect ELISA using commercial typing sera: goat antibodies against mouse immunoglobulins of different isotypes. The vast majority of Ab have a sub-isotype corresponding to the mature immune response of the mouse to protein antigens, namely: IgG1 (6 pcs), IgG2a (7 pcs) and Ig2b (5 pcs). Three Ab have the IgG3 sub-isotype. All L-chains were of the kappa isotype. The activity of antibodies was determined in tests assessing the inhibition of the antiproliferative action of IFN-β on 3 human tumor lines: MCF7 (ATCC HTB-22) breast cancer carcinoma, HT-29 (ATCC HTB-38) colon carcinoma and AGS (ATCC CRL-1739) gastric carcinoma. Analysis of the murine antibodies of a panel of hybridomas for their ability to inhibit the antiproliferative effect of IFN revealed the B16 antibody as a candidate for BsAb construction (Figure S2).

The synthesis of the cDNA coding for the VH and VL variable domains of B16 mAB was performed using total RNA isolated from B16-producing hybridoma cells. Reverse transcription was performed using reverse transcriptase MMuLV (RNase H minus) and oligo (dT18) primer. The synthesis of the second strand of cDNA was carried out using oligonucleotide G-oligo (Table S1) and gene-specific primers complementary to the 5′-end of the antibody constant domain sequences. The amplified and purified fragments of the heavy and light chains were cloned into a linearized pAL2-T vector (Evrogen, Moscow, Russia). In order to determine the consensus nucleotide sequence of the variable domains, at least 10 clones carrying the insert of each of the antibody chain were taken for sequencing. Nucleotide sequence analysis was performed by Chromas, CLC Sequence Viewer and Gene Runner software. As a result, VH and VL consensus nucleotide sequences were determined and compared with the databases of immunoglobulins, IMGT (www.imgt.org, accessed on 19 December 2021), which confirmed their belonging to the genes of mouse immunoglobulins and made it possible to determine the most-related germ lines. Based on the analysis of the primary sequences using the CLC Sequence Viewer, the amino acid sequences of the VH and VL immunoglobulins were determined. Framework (FR) and hypervariable (CDR) regions of variable domains of B16 were established in accordance with the IMGT rules (Figure S1). Mass spectrometric analysis of the molecular weight of the peptide products of mAb hydrolysis confirmed the correctness of the previously established amino acid sequences VL and VH. The presence of peptides corresponding to the constant domains previously determined by isotyping the mAb panel was also proven (data not shown).

### 3.2. Obtaining and Studying the Properties of Chimeric Antibody B16 and Trastuzumab Analogue

**Figure 2:**
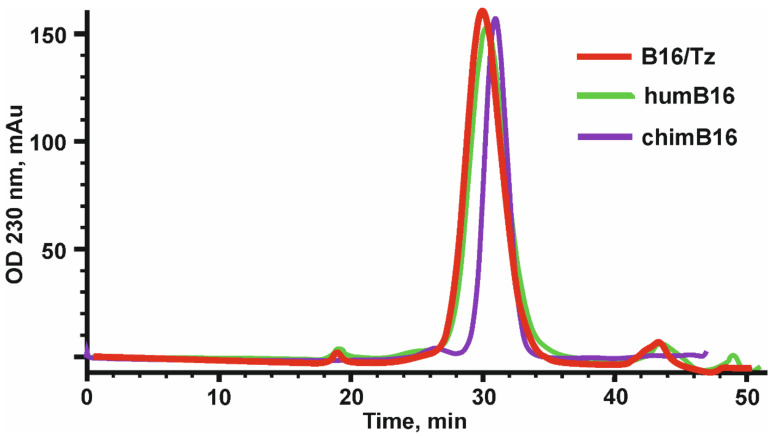
Elution profile of chimeric (chimB16), bispecific antibody (B16/Tz) and humanized (humB16) antibodies, obtained by size-exclusion chromatography on a Superdex 200 10/300 GL column. Initial protein concentration was equal to 0.5–1.1 mg/mL, and the sample volume was 200–500 µl. Protein separation was performed at a 0.4 mL/min flowrate.

The design, preparation and characterization of chimeric antibodies neutralizing IFN-β were carried out as described in Materials and Methods. The L and H chain genes of the chimeric B16 Ab were cloned into the pcDNA3.4 expression vector under the control of the CMV promoter, the thymidine kinase terminator containing the polyadenylation signal and the WPRE element translation enhancer. For the secretion of antibodies into the culture liquid, leader peptides of the B16 antibody were retained (Figure S1). At the 5′-ends of leader peptides, a *Nhe*I restriction site essential for further cloning and Kozak sequence, which is important for RNA efficient translation, were also introduced. For the production of chimeric antibodies, we used a transient expression in CHO cells. After the isolation of antibodies by affinity chromatography, they were analyzed by size-exclusion chromatography (Figure 2).

**Figure 3:**
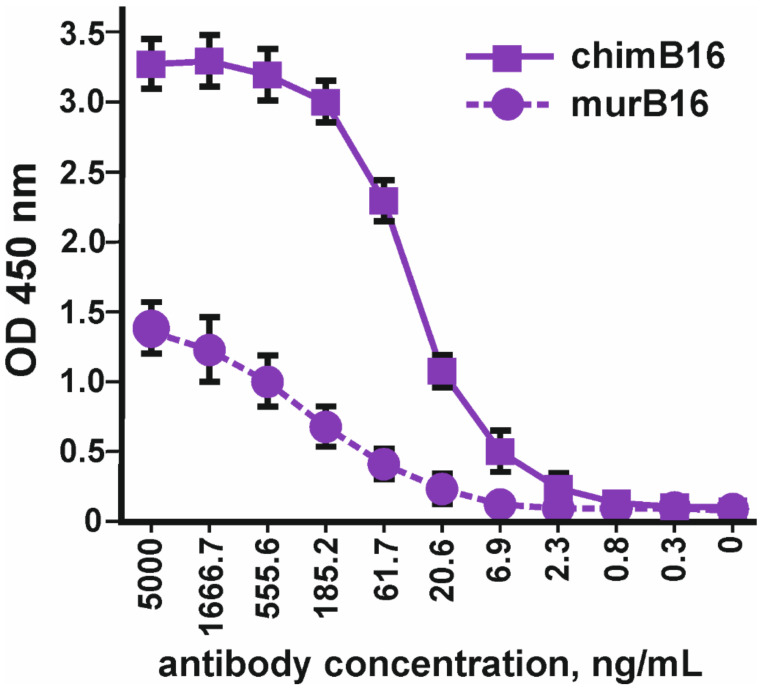
Binding of chimeric antibodies (chimB16) with IFN-β-1a versus murine antibodies (murB16) (Pharmapark, Russia). The error bars indicate means ± SD.

Using the method of indirect ELISA, the binding of the obtained chimeric antibodies to IFN-β (Pharmapark, Russia) was checked in comparison with the prototype mouse antibodies (Figure 3).

It should be noted that the absolute OD values for murine and chimeric antibodies cannot be compared, since different anti-species conjugates were used for the manifestation of binding. The dissociation constant (Kd) of chimeric antibodies (3.2 × 10^−^^9^ M) practically coincides with the Kd of the murine prototype (3.8 × 10^−^^9^ M).

In parallel with the creation of a chimeric antibody against IFN-β, we designed, obtained and characterized the antibody against the ErbB2 receptor (Tz), which is an analogue of Herceptin. The VL and VH nucleotide sequences of antibodies against the ErbB2 receptor were synthesized by overlapping oligonucleotides based on the amino acid sequences of the trastuzumab antibody [37], taking into account the optimization of the codon frequency in the Chinese hamster genome. At the 5′-ends of the VL and VH antibody sequences, the Kozak sequence and leader peptide sequences were placed for the secretion of antibodies into the culture liquid.

### 3.3. Design and Construction of Bispecific Antibodies B16/Tz and Tz/B16

**Figure 4:**
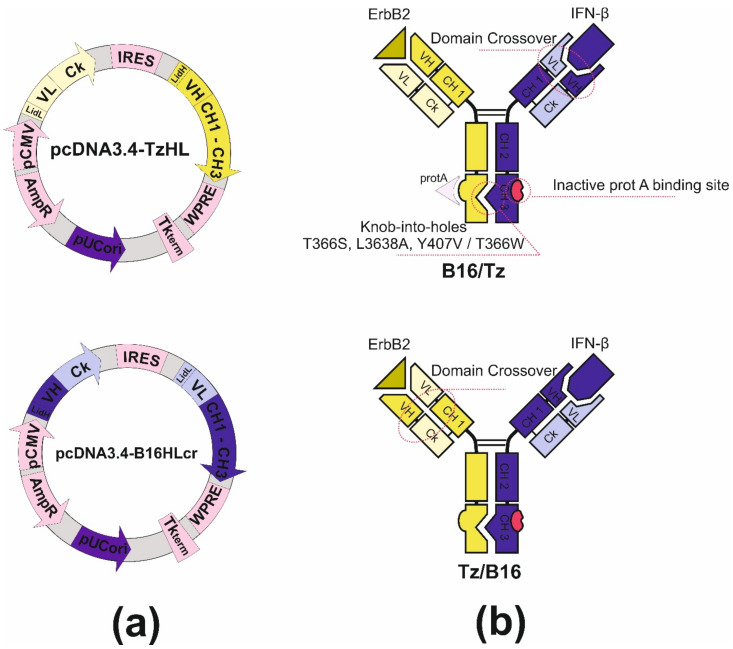
(**a**) Plasmid maps for B16/Tz bispecific antibody production: pcDNA3.4-TzHL and pcDNA3.4-B16HLcr for B16/Tz; (**b**) structure of B16/Tz and Tz/B16 CrossMab bispecific antibodies.

Bispecific antibody construction was started with the introduction of knob-into-holes mutations into IgG1 heavy-chain genes of antibodies by side-directed mutagenesis with primers (Table S3). For the anti-ErbB2 antibody, we made hole substitutions (T366S, L3638A, Y407V) for the chimB16 knob substitution (T366W). Two other mutations, H457R and Y458F, were also introduced in the gene of the B16 heavy chain to enable BsAb gradient purification. The mutant chains were further used for variable domain recombination, which is called crossover. Two CrossMab antibodies, B16/Tz and Tz/B16, (Figure 4b) were constructed differing only by the location of the crossover of variable domains. We have made a crossover of B16 variable domains for B16/Tz and of the Tz arm for Tz/B16. The crossover of variable domains was performed by a series of SOE-PCRs with the use of gene-specific primers (Tables S4 and S5). As a result of cloning, four plasmids were obtained (Table 1). For cell transfection, we combined pcDNA3.4-TzHL to produce a trastuzumab arm of BsAb and pcDNA3.4-B16HLcr to produce a B16HL crossover arm of BsAb (Figure 4a) for B16/Tz biosynthesis, and pcDNA3.4-TzHLcr and pcDNA3.4-B16HL for Tz/B16 biosynthesis, respectively.

### 3.4. Studying the Properties of Bispecific Antibody B16/Tz

**Figure 5:**
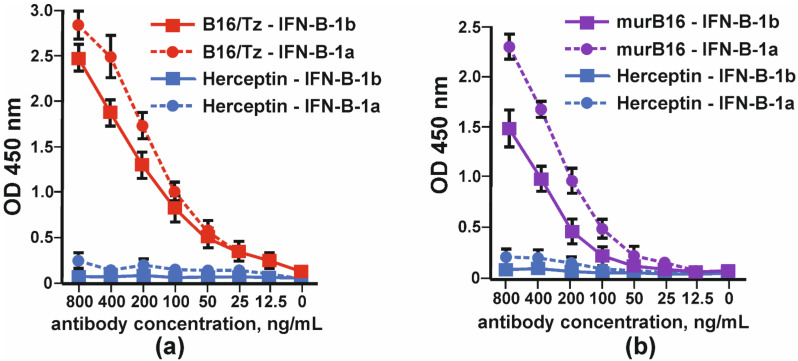
Binding of glycosylated (IFN-β-1a) and non-glycosylated (IFN-β-1b) by: (**a**) CrossMab B16/Tz and (**b**) murine (murB16) antibodies. Herceptin was used as a negative control. The error bars indicate means ± SD.

The binding of bispecific antibody B16/Tz versus murine B16 antibody to IFNβ was tested by indirect ELISA. Two forms of interferon, IFN-β1a and IFN-β-1b, were absorbed on each well’s surface. The anti-ErbB2 antibody (Tz) was used as a negative control. Bispecific antibody B16/Tz, like the prototype mouse B16 antibody, binds to both forms of interferon with greater efficiency to the glycosylated form of IFN-β (Figure 5).

**Figure 6:**
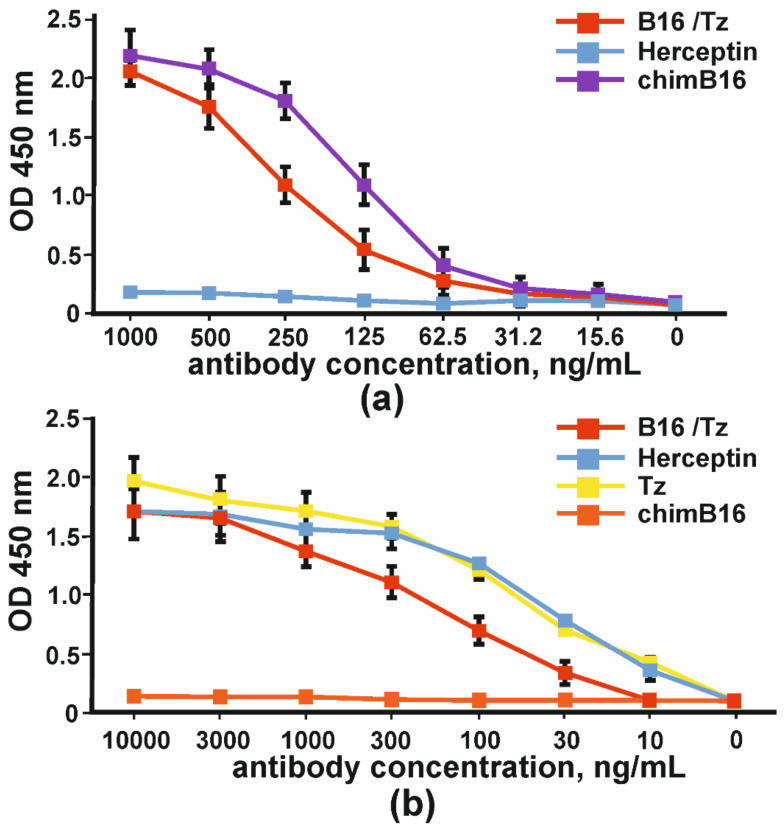
The binding of bispecific antibody B16/Tz, chimeric antibody chimB16, trastuzumab analogue Tz to: (**a**) IFNβ-1a and (**b**) ErbB2^+^SKOV-3 lysates. As negative controls, Herceptin and anti-shiga toxin antibodies were used. The error bars indicate means ± SD.

The comparison of the binding capacity of chimeric antibody (chimB16) and CrossMab B16/Tz to IFNβ-1a in one assay demonstrated the lower potency of the BsAb (Figure 6a). In order to prove that bispecific antibodies bind to the ErbB2 receptor, we absorbed SKOV-3 cell lysates (ErbB2^+^) on plates. The binding curves of B16/Tz, Herceptin and anti-ErbB2 Tz analogue, obtained in this work, are presented in Figure 6. As a negative control, anti-shiga toxin antibody was used. It can be inferred that the binding capacities of Herceptin and its analogue may be considered equal, while CrossMab antibody B16/Tz binds with less capacity (Figure 6b).

**Figure 7:**
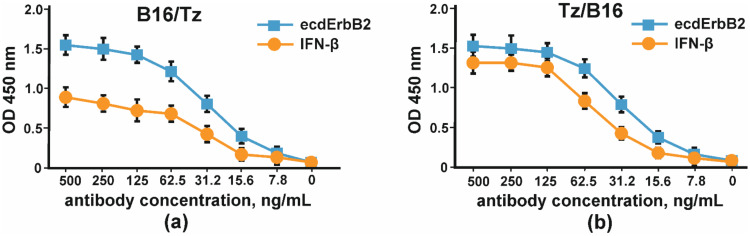
The binding of IFNβ and the extracellular part of ErbB2 by CrossMab antibodies: (**a**) B16/Tz and (**b**) Tz/B16. The error bars indicate means ± SD.

The binding of CrossMab antibodies B16/Tz and Tz/B16 was proven by indirect ELISA with IFNβ and the extracellular part of ErbB2 (Figure 7).

**Figure 8:**
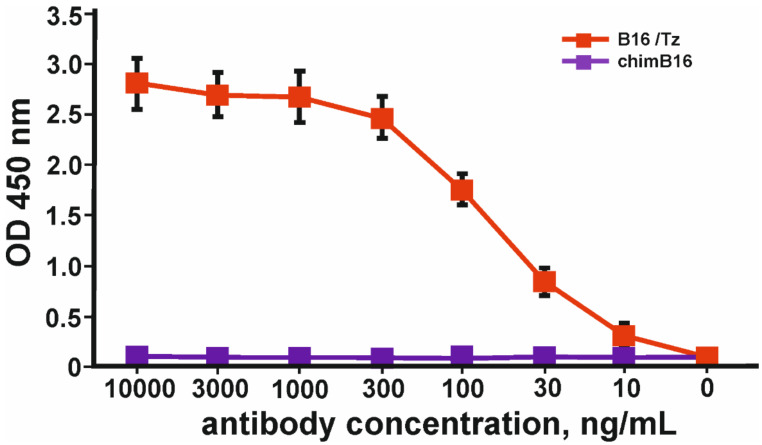
Binding curves of simultaneous binding of two antigens: IFN-β and ecdErbB2 in sandwich ELISA. The error bars indicate means ± SD.

The simultaneous binding of IFN-β and ErbB2 was proven by sandwich ELISA according to the scheme shown in Figure S3. In order to prove the ability of B16/Tz to bind two different antigens, which is essential for bispecific antibodies, we proposed a sandwich ELISA system with two antigens. IFN-β was used to immobilize CrossMab through its anti-interferon arm, and biotinylated-ecdErbB2 recombinant protein was used to detect BsAbs through their ErbB2 binding activity. The result of the experiment is shown in Figure 8.

### 3.5. Humanization of Antibody B16

**Figure 9:**
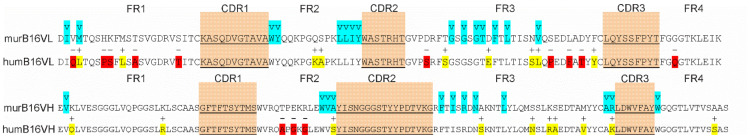
Amino acid sequences of murB16 and hum B16 variable domains. CDRs are underlined, blue for Vernier zones, red and yellow for substitutions.

For murine antibody humanization, the CDR-grafting method was chosen. We decided to use the IMGT database containing human germline Ig genes for the Ig FR-donor search. Among a wide diversity of sequences, we found IGHV3-23*04 and IGKV1-9*01 genes with a homology degree of 80% and 65% to murine B16 heavy- and light-chain variable domains, respectively. The comparison of humanized and murine variable domain sequences resulted in discovering 10 critical substitutions and 9 conservative ones in light-chain variable domain and 3 critical substitutions and 10 conservative ones in heavy-chain variable domain. The analysis of Vernier zone residues showed no critical replacements there; therefore, no reverse mutations were introduced (Figure 9).

To make sure that the proposed method of humanization by CDR-grafting did not significantly affect the structure of the antibody, we created 3D-structure models of murine and humanized B16 variable domains using Rosetta Antibody web service. A few steps were performed: (1) searching for antibodies with known spatial structure and similarity to humB16 amino acid sequences, (2) building initial models using conservative template fragments and humB16 VL and VH query sequences and (3) improving the initial models by selecting optimal conformations of the main chain and amino acid residues. Then the obtained models were optimized by taking into account the influence of the solvent, and molecular dynamics calculations were performed in Gromacs and Charmm36 force field. The solvent was H_2_O and the parameters: 300 °K, physiological concentrations of NaCl, simulation length 10 ns. After that MD trajectories were subjected to cluster analysis, the most representative of those were selected as final models.

**Figure 10:**
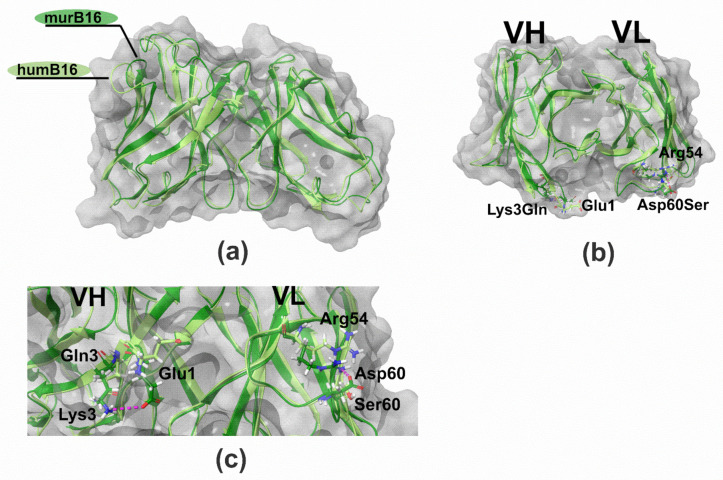
Structure of B16 wild-type and humanized variable domains: (**a**) superimposed structures of humB16 and murB16 variable domains; (**b**) location of K3Q and D60S mutations on superimposed structures of humB16 and murB16 variable domains, view from the top; (**c**) change of position of G1 (VH) and R54 (VL) after introducing K3Q (VH) and D60S (VL) mutations in variable domains. Light green and dark green colors of main chains were used for murB16 and humB16 variable domains, for side chains red, blue, white and green colors were used for oxygen, nitrogen, hydrogen and carbon atoms. Pink dashed line – hydrogen bonds and salt bridges.

The comparison and superposition of murine and humanized variable domain models showed no crucial displacement in the backbone chain (Figure 10a). In particular, some mutations like H8P and R44G led to the noticeable local displacement of the backbone chain with no effect on the structure of the antibody paratope. More detailed analysis of models with the research of the positioning of residues made it possible to formulate a number of recurrent substitutions in framework regions of the humanized antibody. Two mutations, K3Q in the heavy chain and D60S in the light chain, may lead to surface changes of the surroundings of the paratope (Figure 10b). In murine B16, heavy-chain K3 forms a salt bridge with E1, and the replacement of lysine by glutamine disrupts the interaction between those two charged residues. As a result, the glutamate side chain changes its position toward the paratope surface. The replacement of Asp60 by Ser in light chain makes impossible the formation of an interaction with Arg54, which changes the orientation of this CDR2 residue (Figure 10c). Both mutations K3Q and D60S change the location of charged residues near the environment of the paratope, which may influence negatively on the Kd value of the antibody.

**Figure 11:**
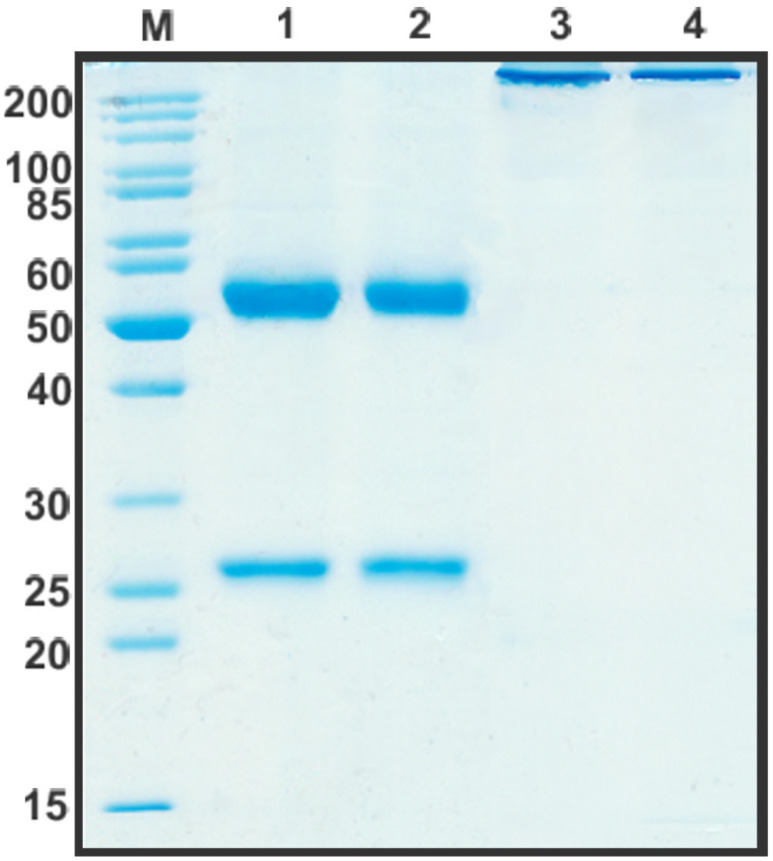
SDS-PAGE in 12% gel of purified by affinity chromatography chimB16 (lane 1, 3) and humB16 antibodies (lane 2, 4). Lane 1, 2—reducing conditions, lane 3, 4—non-reducing conditions.

After the construction of humB16 genes and cloning them into the expression vector, both humanized and murine B16 antibodies were produced in CHO cells and purified, and their properties were studied. The yields of purified proteins were measured and counted up to 330 mg/L for chimB16 and 353 mg/L for humB16. Elution profiles obtained by the size-exclusion chromatography of humB16 and chimB16 have similar character, with the retention time of both antibodies ranging from 31–32 min, corresponding to the monomeric form of IgG with a calculated molecular weight 162–165 kDa (Figure 2). The purity of the obtained antibodies is also confirmed by SDS-PAGE (Figure 11); under non-reducing conditions, immunoglobulins migrate as a single band, but after disulfide bond reduction with mercaptoethanol, both humanized and murine antibodies migrate as two bands corresponding to 25 kDa light and 50 kDa heavy chains.

**Figure 12:**
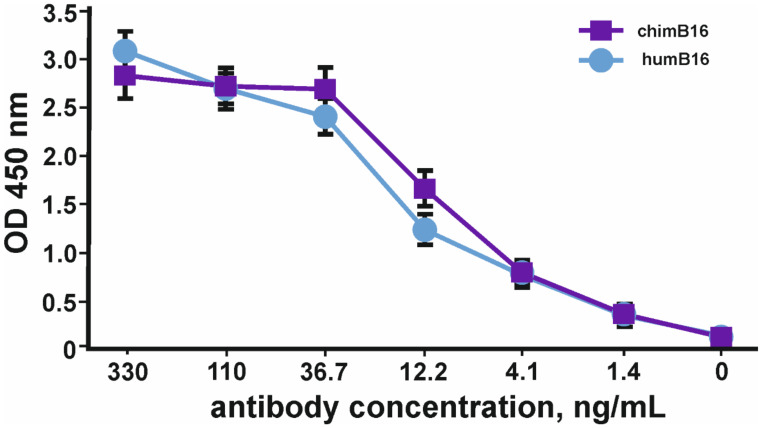
IFN-β binding curves by humB16 and chimB16 antibodies. The error bars indicate means ± SD.

Binding curves to IFN-β of both antibodies (Figure 12) characterize them as antibodies with a high degree of affinity, which is confirmed by Kd values 3.2 × 10^−^^9^ M for chimB16 and 3.3 × 10^−^^9^ M for humB16. 

### 3.6. Study of the IFN-β Neutralization Capacity of Chimeric ChimB16 and Bispecific B16/Tz Antibodies

**Figure 13:**
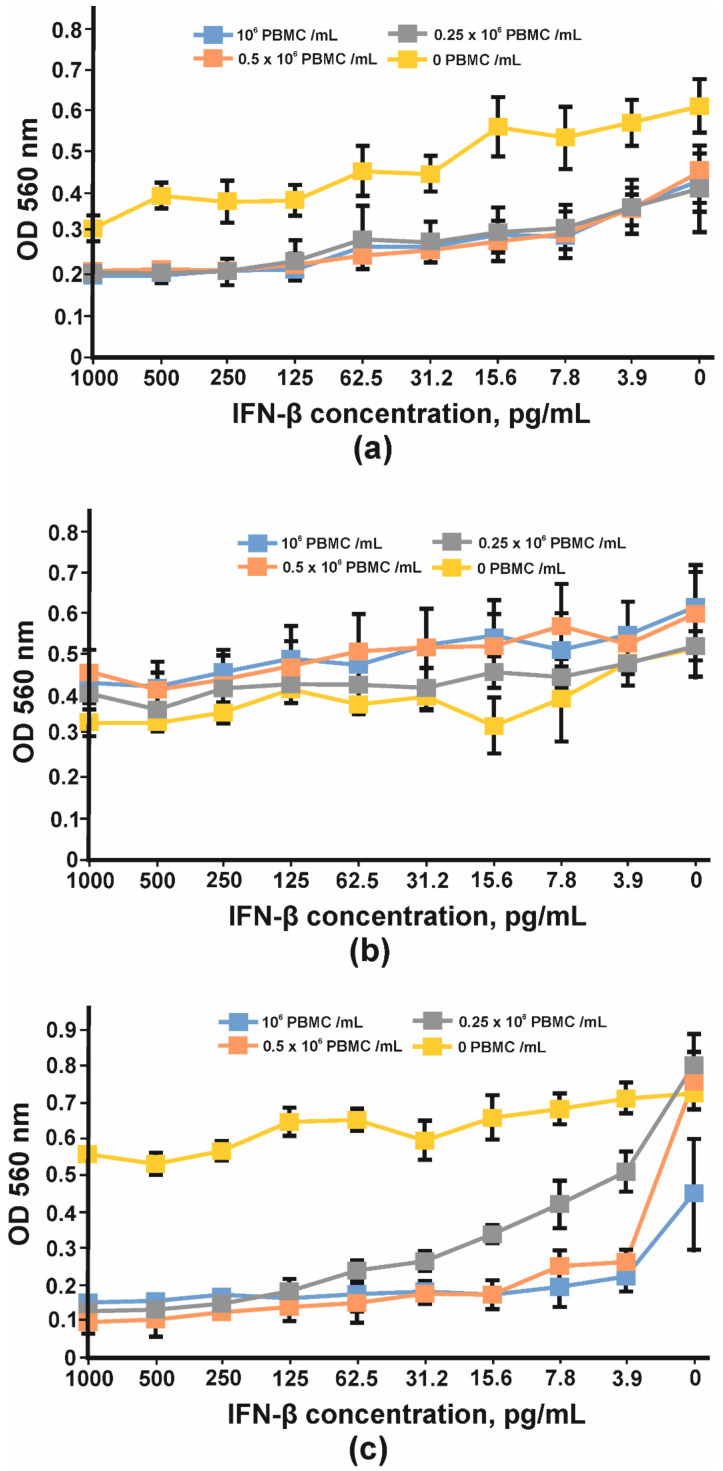
Inhibition of cell proliferation by IFNβ and PBMC: (**a**) SKOV-3, (**b**) BT474 and (**c**) HT29 cell lines. The error bars indicate means ± SD.

In order to find the appropriate cell line for IFN-β antiproliferative assay, three cell lines, HT29, SKOV-3 and BT474, were tested. After four days of cultivation with serial dilutions of IFN-β and PBMC, the viabilities of the cells were determined using an MTT test. Neither interferon nor PBMC had any impact on BT474 cells (Figure 13b). A more pronounced antiproliferative effect of IFNβ was observed for SKOV-3 cells (Figure 13a); however, the addition of PBMC decreased the slope of the curve and the maximum OD value so that the detection of the growth inhibition effect could be problematic. No significant antiproliferative effect mediated by IFN-β was observed for HT29 cells; nevertheless, the combination of interferon and PBMCs greatly decreased HT29 cell growth. The viability of HT29 cells cultivated without interferon did not change substantially with the addition of 25,000 cells (0.25 × 10^6^ PBMC/mL) or 50,000 cells (0.5 × 10^6^PBMC/mL) per well but decreased by at least 50% when 100,000 PMBCs were added (10^6^ PBMC/mL) (Figure 13c). That is why 100,000 PMBCs/well were unsuitable for further antibody IC measurement. It should be noted that interferon dilutions for the remaining two curves decreased more for 0.5 × 10^6^ PBMC/mL than for 0.25 × 10^6^ PBMC/mL. The maximum antiproliferative effect for 0.5 × 10^6^ PBMC/mL was observed at 15.62 pg/mL of IFN-β and did not change significantly at higher IFN-β concentrations.

**Figure 14:**
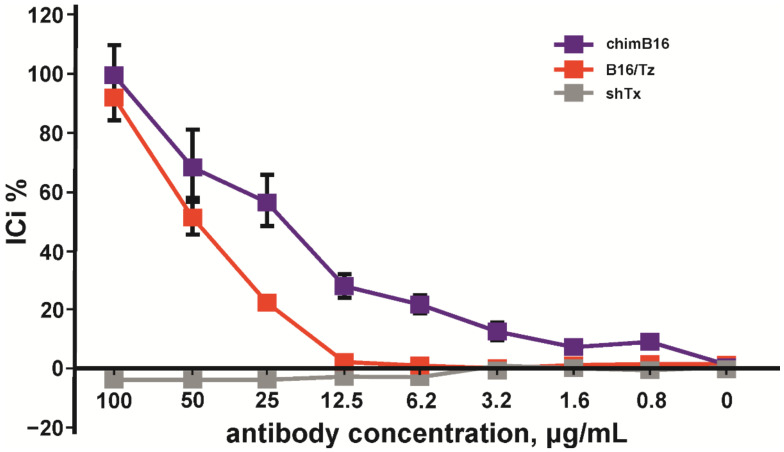
Inhibition of IFN-β antiproliferative activity by chimB16 and B16/Tz antibodies, shown on HT 29 cells. An anti-shiga toxin antibody was used as a negative control. The error bars indicate means ± SD.

Considering the above-mentioned facts, we chose the HT29 cell line for IC determination. A total of 100 pg/mL of IFN-β, 0.5 × 10^6^ PBMC/mL, was added to adherent cells. The inhibition of the IFN-β antiproliferative effect by anti-IFN-β antibodies was performed in a concentration range of 0.5–100 μg/mL. The inhibition of the antiproliferative activity of IFN-β was observed for chimB16 starting from 3.1 μg/mL and for CrossMab B16/Tz from 25 μg/mL. The IC50 values for both antibodies were determined and turned out to be 21.8 μg/mL for B16 and 49.3 μg/mL for B16/Tz (Figure 14). As a negative control, anti-shiga toxin antibody was used. No inhibition effect on IFN-β was observed for the control antibodies. Taking all that is mentioned above into account, we can state that both B16/Tz and B16 possess the ability to neutralize IFN-β.

## 4. Discussion

The main emphasis in the analysis of a panel of hybridomas producing antibodies against IFN-β was focused on the Ab affinity and their ability to neutralize the antiproliferative effect of IFN-β. The main goal of our work was to create a vehicle for IFN-β delivery to the site of ErbB2-associated tumor cells. At the same time, IFN-β during transportation should not react with IFNAR receptors and cause undesirable side effects. Therefore, the arm of BsAb responsible for cytokine binding must be able to neutralize IFN-β activity. Analysis of mouse antibodies from a panel of hybridomas by their affinity to IFN-β and their ability to inhibit its antiproliferative effect revealed the candidate antibody B16. The data obtained indicate that Ab B1–B21 have a high affinity for non-glycosylated and glycosylated forms of recombinant human IFN-β (B1–B21 Kd values range within 0.4–61.0 nM).

Based on the results of the analysis of the panel of hybridomas, the B16 antibody was selected. The dissociation constant of murB16 is in the nanomolar range (3.8 × 10^−^^9^ M), and murB16 has the best neutralizing properties in relation to IFN-β, which is a good prerequisite for the development of a bispecific antibody on itsbasis.

Total RNA was isolated from B16-producing hybridoma cells, and reverse transcription was performed. Immunoglobulin cDNAs were obtained and cloned in order to establish consensus sequences of the variable domains of the H- and L-chains of B16 mAb. The sub-isotypes of the Ab B16 chains were also confirmed. FR and CDR regions of the variable domains of B16 were established in accordance with the Kabat rules (Figure S1). Mass spectrometric analysis of the molecular weight of the peptide products of mAb hydrolysis confirmed the correctness of the previously established amino acid sequences VL and VH (Figure S1). The presence of peptides corresponding to the constant domains previously determined by isotyping the mAb panel was also proven. In the first step, the VH and VL of the murine B16 antibody were combined with the constant domains of human antibodies and cloned into the pcDNA3.4 vector for production in a mammalian cell line. The produced chimeric antibodies were tested for binding to IFN-β and for the ability to inhibit its antiproliferative effect in comparison with the prototype mouse antibody. The chimeric antibody has been shown to have the same characteristics as the murine antibody. The dissociation constant (Kd) of chimeric antibodies (3.2 × 10^−^^9^ M) practically coincides with the Kd of the murine prototype (3.8 × 10^−^^9^ M). Based on chimeric B16 antibody and antibodies against the ErbB2 receptor Tz (Herceptin analogue), obtained and tested for binding both to lysates of ErbB2-positive cell lines and to the recombinant extracellular domain of the ErbB2 receptor, we designed a bispecific antibody capable of binding to both IFN and ErbB2 receptor.

Among a large number of BsAb formats, CrossMab stands out due to a few noticeable characteristics. First, its structure highly resembles IgG in the number of domains and its composition. CrossMab antibodies consist of two distinct heavy and two distinct light chains with total Mw about 160 kDa, Y-shaped form of the molecule and IgG constant and variable domains. Because of the high molecular weight and availability of the Fc-fragment, which interacts with neonatal Fc-receptor, it is supposed that the serum half-life of CrossMab antibodies would be the same as for most antibodies, around 21 days [38]. Second, CrossMab may have the ability of mediating Fc-mediated antibody dependent cellular cytotoxicity, antibody dependent cellular phagocytosis and complement system activation [39], characteristics lacked by small-size formats like BiTE or DART [40]. Finally, a lack of peptide linkers or other potent immunogenic sequences and the high stability of those molecules makes CrossMab platform very attractive for bispecific antibody development.

The construction of CrossMab bispecific antibodies includes a few steps. First is the introduction of knob-into-holes mutations into the genes of heavy chains of two monoclonal antibodies prototypes. The co-expression of two heavy chains may result in the formation of a heterodimer and two homodimers, which do not form bispecific antibodies. That happens because of the identity of CH3 domain surfaces whose role in heavy chain dimerization is more than essential. The introduction of knob-into-holes mutations might solve that problem with high-efficiency heterodimer formation. To completely exclude the presence of the chimB16 antibody, we also introduced H457R and Y458F mutations into its heavy-chain gene. Those mutations dramatically decrease the sorption of antibodies on protein A resins and open up the possibility of the separation of the bispecific antibody and monoclonal antibodies by pH-gradient elution [41]. As a result of introducing the mentioned mutations, we managed to obtain bispecific antibodies with a purity degree higher than 95% (Figure 2 and Figure 11).

The second complication that should be overcome is the light-chain mispairing. The molecular architectures of interdomain interfaces of light and heavy chains in both arms of both antibodies that constitute BsAb are identical, which results in the formation of non-functional pairs of heavy-light chains. It is possible to make those interfaces different by introducing specific substitutions on interfaces between variable heavy and variable light and between constant heavy and light domains. Mutations described by Lewis (heavy chain Q38R, S176W, L135Y, light chain Q39Y, F174G, H172A) [42] and Bönisch (heavy chain F118S/A/V, T129R, light chain A114L, K120D) [43] disfavor the pairing of mutant chains with wild-type chains, thus introducing the aforementioned substitutions into the genes of one of two antibodies can solve this complication. An alternate approach to make interfaces different, termed domain crossover, is described by Schaefer [44]. This term is used in relation to the rearrangement of heavy- and light-chain domains in one arm of two antibodies forming the bispecific antibody. We designed two versions of CrossMab antibodies. In the first one, B16/Tz, we leave the heavy and light chain interfaces of anti-ErbB2 antibody unmodified, but for the B16 antibody, we made a rearrangement of variable domains forming new heavy chain consisting of heavy chain constant domains and light chain variable domain and new light chain made of constant light and variable heavy domains. For the second version of CrossMab Tz/B16, a crossover approach was applied in relation to anti-ErbB2 antibody variable domains. In order to produce CrossMab antibodies, four different genes should be cloned into an appropriated vector system. We decided to modify the pcDNA3.4 vector with multiple cloning site linkers to construct a bi-plasmid expression system consisting of two bicistronic plasmids, each carrying half of the genes of a bispecific antibody. After cloning light- and heavy-chain genes under the control of the CMV promoter and TK terminator, we introduced encefalomiocarditis virus IRES-element between them. That regulatory element in our constructs is essential for heavy-chain translation, as long as heavy-chain genes are located downstream of light-chain genes in mRNAs (Figure 4a). The described vector design ensures the comparable transcription and translation levels of both chains in each of the arms of the bispecific antibody.

Chimeric antibodies are usually generated by fusing human immunoglobulin constant domains to variable domains of a selected mouse hybridoma, which produce monoclonal antibodies specific to the target molecule. As a result, chimeric antibodies contain about 33% murine amino acid sequences. That may cause human anti-murine antibody response (HAMA response), decreasing serum half-life and therapeutic efficacy. To mitigate the HAMA-response, chimeric antibodies may be humanized. A few strategies of antibody immunogenicity reduction are proposed by different authors: humanization by antibody resurfacing, humanization based on human string content optimization, superhumanization, SDR-grafting and CDR-grafting methods. While most of the named methods rely on complex computational calculations, CDR-grafting may be performed by the manual annotation of CDRs and FRs and replacing xenogeneic frameworks by homological sequences obtained from human Ig genes.

After identifying appropriate FR-donors, IGHV3-23*04 and IGKV1-9*01 genes, we replaced their CDR-regions according to the Kabat numbering system by murine B16 CDRs and compared murine and humanized variable domain sequences. The comparison enlightened 10 critical substitutions and 9 conservative ones in the light chain and 3 critical substitutions and 10 conservative ones in the heavy chain that could be explained by the higher homology degree of heavy chains. Although FR-regions do not play a crucial role in antibody–antigen interactions, some FR-residues called Vernier zone residues are very important for CDR loop positioning. Their inaccurate replacement may result in dramatic antibody affinity loss. We identified and analyzed Vernier zone residues and found only four irrelevant amino acid replacements, which were left without recurrent mutations.

In order to figure out whether the spatial conformation is affected by CDR-grafting, 3D-structure models of humanized and murine variable domains were built. First to note, the comparison of the 3D-structure models of murine and humanized B16 made clear that the replacement of frameworks did not dramatically affect the positioning of the backbone chain (Figure 10a). Although few mutations led to the local displacement of the backbone chain, the paratope structure was unaffected. However, after detailed residue positioning analysis, two mutations (K3Q in VH and D60S in VL, Figure 10b) were found to possibly affect the structure of the paratope surface surroundings. In particular, K3Q mutation changes the position of E1 closer to the paratope cavity. Another replacement D60S changes the orientation of R54, a CDR2 residue (Figure 10c). Both mutations may affect the interaction of humanized B16 antibody with interferon, which is why we decided to retain murine residues K3 and D60 in the humanized version of the B16 antibody. All of those described actions and the described approach were sufficient to retain a humanized humB16 antibody affinity with a minimal increase in Kd value from 3.2 × 10^−^^9^ for chimB16 to 3.3 × 10^−^^9^ for humB16. Cytokines are small protein molecules (10–25 kDa) playing an important role in immunological processes and cell signaling; however, their excess could cause some pathological conditions e. g. rheumatoid arthritis or cytokine storm. Anti-cytokine antibodies may increase cytokine clearance and alleviate symptoms, but neutralization is essential for blocking undesirable effects [45]. The potency to neutralize antigens is one of the most important characteristics of the antibodies. The neutralization ability is vital for viral infection prevention [46] or antitoxin effect mediation [47]. In our work, we constructed anti-ErbB2 and anti-IFN-β CrossMabs as a vehicle for endogenous and exogenous IFN-β for cancer treatment. Despite outstanding antitumor activity of IFN-β, the use of that cytokine is limited due to side effects and toxicities. One way to overcome this challenge is to deliver it directly to the tumor site by linking it to an antibody. Lee and coauthors [48] described and characterized an immunocytokine protein based on trastuzumab and IFN-β linked by a peptide linker. That recombinant protein has superior antitumor activity compared to both trastuzumab and IFN-β, showing great antiproliferative characteristics both on ErbB2^+^ YCC-38, NCI-N87 and YCC-19 cell lines and in xenograft murine models. Though that strategy of cytokine delivery is rational and effective, there is no guarantee of the absence of several side effects in clinical practice due to the fact that the IFN-β in the composition of such a molecule might not be neutralized and can interact with IFNAR during transportation and blood circulation. The neutralization of interferon with one arm of anti-IFN-β bispecific antibody in a complex may resolve that complication, while the second arm is responsible for the targeting of the complex. Since IFN-β plays an essential role in human immunity, the binding and the depletion of endogenous interferon by B16/Tz antibody may result in several side effects. Therefore, B16/Tz—IFN-β complex formation should be established before administration by mixing equimolar amounts of interferon and BsAb. The high affinity of the BsAb may guarantee the formation of the complex with high effectiveness, and the exogenous interferon molecules dissociated during blood circulation may be replaced by endogenous ones without substantial IFN-β blood concentration alterations. Thus, the proposed approach in our work may be used for the safe delivery of IFN-β to tumors.

To implement such a strategy before starting CrossMab construction, it is necessary to be sure of IFN-β neutralization, using the antibody constituing CrossMab. It is also important to be secure that the ability to neutralize IFN-β will also remain after domain crossover performance. For this purpose, we used a method based on the registration of cell-proliferation suppression by IFN-β in the presence of peripheral blood mononuclear cells (PMBC). After the preliminary analysis of HT29, SKOV-3 and BT474 cell lines, we chose HT29 cells because of their high sensitivity to IFN-β, an important point for neutralization studies (Figure 12). For further experiments, we added serial dilutions of tested antibodies to HT29 cells and incubated them with fixed concentration of interferon and PBMCs. It was demonstrated that B16/Tz and B16 are able to bind and neutralize IFN-β with IC50 values of 21.8μg/mL and 49.3μg/mL, respectively (Figure 13). Since CrossMab antibody is composed of two different arms, B16/Tz lacks one of two anti-interferon paratopes. This fact explains the decreased avidity of CrossMab compared to the monoclonal antibody and, obviously, the augmented value of IC50. We can also state that the domain crossover performed for B16 did not result in the substantial loss of avidity or ability to neutralize IFN-β, an important condition for the safe delivery of interferon to solid tumors.

## 5. Conclusions

Based on the neutralizing antibody B16 to IFN-β and the trastuzumab analogue specific to the ErbB2 receptor, we obtained a bispecific antibody of the CrossMab format with knob and hole mutations in the CH3 domain of H chains. The obtained B16/Tz BsAb was shown to bind and neutralize IFN-β, simultaneously binding the ErbB2 receptor in tumor cell lysates and in the form of a recombinant extracellular domain. Thus, BsAb can be used as a component of the immunocytokine complex for the delivery of IFN-β to ErbB2-associated tumor cells, which would avoid the manifestation of side effects when IFN-β is administered as a single therapeutic. It is planned to test this approach in the therapy of ErbB2-associated tumors in animal models.

## Figures and Tables

**Table 1:** Constructed plasmids for CrossMab production.

CrossMab	Plasmid	Heavy Chain	Light Chain
B16/Tz	pcDNA3.4-TzHL	Anti-ErbB2 hole	Anti-ErbB2
	pcDNA3.4-B16HLcr	B16 knob with domain crossover	B16 with domain crossover
Tz/B16	pcDNA3.4-TzHLcr	Anti-ErbB2 hole with domain crossover	Anti-ErbB2 with domain crossover
	pcDNA3.4-B16HL	B16 knob	B16

## Data Availability

The data that support the findings of this study are available from the corresponding author upon reasonable request.

## Supplementary Materials

The following are available online at https://www.mdpi.com/article/10.3390/biom11121915/s1, Figure S1: Heavy- and light-chain chimB16 antibody amino acid sequences, Figure S2: The analysis of IFN-β antiproliferative effect inhibited by antibodies produced by hybridomas B1–B21. Figure S3: Scheme of sandwich ELISA with the simultaneous binding of two antigens: IFN-β and ecdErbB2, Table S1–S6 is a list of oligonucleotides used for the present work.
