# Supplementary material for: Bispecific Antibodies for IFN-β Delivery to ErbB2+ Tumors
Source: Biomolecules. 2021 Dec 20;11(12):1915. doi: 10.3390/biom11121915 (PMC8699518; doi:10.3390/biom11121915)

Supplementary Material to the manuscript

Table S1. Nucleotide sequences of the primers used for amplification of the heavy and light chains (VH and VL) of musB16.

Oligonucleotide	Oligonucleotide sequence 5 '- 3'
1NesHuG	GTCCTTGACCAGGCAGCCC
2NesHuG	GATGGGCCCTTGGTGGA
1NesHuKap	ATTTCAACTGCTCATCAGATGG
2NesHuKap	GATGAAGACAGATGGTGCAGC
G-oligo	AAGCAGTGGTATCAACGCAGAGTACGCrGrGrG

Table S2. Nucleotide sequences for the synthesis of chimeric B16 chains.

Oligonucleotide	Oligonucleotide sequence 5 '- 3'
B16LidKapKN	AGGGAAGCTAGCGCCACCATGGAGACACATTCTCAGGTCTTTGTATAC
KapF10IRev	CAGCCACAGTCCGTTTTATTTCCAGCTTGGTCC
LkcF	AAACGGACTGTGGCTGCACCATCTGTCTTC
B16LidIgG1KN	AGGGAAGCTAGCGCCACCATGAATTTCGGGCTCAGCTTGATTTTC
BH16VHRevBsp	AGACCGATGGGCCCTTCGTGCTGGCTGCAGAGACAGTGACCAGAG

Table S3. Nucleotide sequences for the synthesis of variable domain of H-chain of Tz- analog antibody.

Oligonucleotide	Oligonucleotide sequence 5 '- 3'
TzHF1	CCAAAGTGCCCAAGCAGAGGTG**CAGCTG**GTGGAGTCTGGAGGAG
TzHR1	GCCTCAGGGAGCCGCCGGGCTGCACCAGGCCTCCTCCAGACTCC
TzHF2	CGGCTCCCTGAGGCTGTCCTGCGCCGCTTCCGGCTTCAACATCAAGGA
TzHR2	GGCCTGCCTCACCCAGTGGATGTAGGTGTCCTTGATGTTGAAGCC
TzHF3	GGGTGAGGCAGGCCCCTGGCAAGGGTCTCGAATGGGTCGCAAGGA
TzHR3	GGCGTACCTGGTGTAGCCGTTGGTGGGGTAGATCCTTGCGACCCATTC
TzHF4	CTACACCAGGTACGCCGACTCCGTGAAGGGCAGGTTCACCATCT
TzHR4	GTAGGCGGTGTTCTTGGAGGTGTCAGCAGAGATGGTGAACCTGCC
TzHF5	CAAGAACACCGCCTAC**CTGCAG**ATGAACTCCCTGAGGGCCGAGGA
TzHR5	CCCCATCTTGAGCAGTAGTAAACTGCTGTGTCCTCGGCCCTCAGG
TzHF6	CTGCTCAAGATGGGGCGGCGACGGCTTCTACG**CCATGG**ACTACT
TzHR6	GACACGGTCACCAGGGTGCCTTGGCCCCAGTAGTCCATGGCGTAG
TzHF7	CCTGGTGACCGTGTCCTCCGCAAGCACGAA**GGGCCC**ATCAAAA
TzHR7	TTTTGATGGGCCCTTCGTT

Table S4. Nucleotide sequences for the synthesis of variable domain of the L-chain of Tz-analog antibody.

Oligonucleotide	Oligonucleotide sequence 5 '- 3'
TzLF1	CTGGTGCTCATGGGGACATCCAGATGACCCAGTCTCCATCC
TzLR1	CGGTCGCCCACGGAGGCGGACAGGGAGGATGGAGACTGGGTC
TzLF2	CTCCGTGGGCGACCGAGTGACCATCACATGCAGGGCCTCCCAGG
TzLR2	TGATACCATGCCACGGCGGTGTTCACGTCCTGGGAGGCCCTG
TzLF3	CCGTGGCATGGTATCAACAGAAGCCCGGCAAAGCTCCCAAACTG

TzLR3	TACAGGAAGCTTGCAGAGTAGATCAGCAGTTTGGGAGCTTTGC
TzLF4	CTGCAAGCTTCCTGTACTCCGGCGTGCCCTCCAGGTTCTCTGGCTC
TzLR4	GTCAGGGTGAAGTCGGTTCCACTCCTGGAGCCAGAGAACCTGG
TzLF5	CCGACTTCACCCTGACCATCTCCTCTCTGCAGCCCGAAGACT
TzLR5	GTAGTGCTGCTGGCAGTAGTAGGTGGCAAAGTCTTCGGGCTGCAG
TzLF6	CTGCCAGCAGCACTACACCACCCCTCCCACCTTCGGACAAGGG
TzLR6	CAGCCACAGTCCGTTTGATCTCGACTTTGGTCCCTTGTCCGAAGGTG

Table S5. Nucleotide sequences for "knob-into-holes" site-directed mutagenesis.

**Oligonucleotide**	**Oligonucleotide sequence 5 '- 3'**
HTR1	CTTTGACGGCGCAGGACAGGCTGACCTGGTTCTTGGTC
HTF1	CTGTCCTGCGCCGTCAAAGGCTTCTATCCCAGCG
HTR2	GAGCTTGCTAACGAGGAAGAAGGAGCC
HTF2	CTTCTTCCTCGTTAGCAAGCTCACCGTG
KTR1	ACCAGGCACCACAGGCTGACCTGGTTC
KTF1	TCAGCCTGTGGTGCCTGGTCAAAGGCTTC
StTyR1	TCTGCGTGAAGCGGTTGTGCAGAGCCTCATGCATCAC
StTyF1	GCACAACCGCTTCACGCAGAAGAGCCTCTCCCTGTC
UniHcdcR	CGATGGGCCCTTCGTGCTGGCGGAGGATTTTATTTCCAGCTTGGTCCC
B16LcdcR	AAGACAGATGGTGCAGCCACGCTGGCTGAGGAGACGGT
UniKapdcF	GTGGCTGCACCATCTGTCT

Table S6. Nucleotide sequences for recombination of heavy and light chain variable domains.

**Oligonucleotide**	**Oligonucleotide sequence 5 '- 3'**
CMVF	CGCAAATGGGCGGTAGGCGTG
pcDNAR	CAACATAGTTAAGAATACCAGTC
UniHcdcR	CGATGGGCCCTTCGTGCTGGCGGAGGATTTTATTTCCAGCTTGGTCCCC
B16LcdcR	AAGACAGATGGTGCAGCCACGCTGGCTGAGGAGACGGT
UniKapdcF	GTGGCTGCACCATCTGTCT
TzHVdcR	GAAGACAGATGGTGCAGCCACGCTGGCGGAGGACACGGT
TzLVdcR	AAGACCGATGGGCCCTTCGTGCTGGCGGAGGATTTGATCTCGACTTTGG

Figure S1. Heavy and light chain chimB16 antibody amino acid sequences.

chimB16 Light chain sequence

Secretory signal — Light chain variable domain

*METHSQVFVYMLLWLSGVEG*DIVMTQSHKFMSTSVGDRVSITCKASQDVGTAVAWYQQKPGQSPKLLI

CDR1

Light chain variable domain

YWASTRHTGVPDRFTGSGSGTDFTLTISNVQSEDLADYFCLQYSSFPYTFGGGTKLEIKRTVAAPSVF

CDR2 — CDR3

Kappa-chain constant domain

IFPPSDEQLKSGTASVVCLLNNFYPREAKVQWKVDNALQSGNSQESVTEQDSKDSTYSLSSTLTLSKA

DYEKHKVYACEVTHQGLSSPVTKSFNRGEC

chimB16 Heavy chain sequence

Secretory signal — Heavy chain variable domain

*MNFGLSLIFLVLVLKGVLC*EVKLVESGGGLVQPGGSLKLSCAASGFTFTSYTMSWVRQTPEKRLEWVA

CDR1

Heavy chain variable domain

YISNGGGSTYYPDTVKGRFTISRDNAKNTLYLQMSSLKSEDTAMYYCARLDWVFAYWGQGTLVTVSAA

CDR2 — CDR3

IgG1 heavy chain CH1 domain

STKGPSVFPLAPSSKSTSGGTAALGCLVKDYFPEPVTVSWNSGALTSGVHTFPAVLQSSGLYSLSSVV

IgG1 hinge region

TVPSSSLGTQTYICNVNHKPSNTKVDKRVEPKSCDKTHTCPPCPAPELLGGPSVFLFPPKPKDTLMIS

IgG1 heavy chain CH2 domain

RTPEVTCVVVDVSHEDPEVKFNWYVDGVEVHNAKTKPREEQYNSTYRVVSVLTVLHQDWLNGKEYKCK

IgG1 heavy chain CH3 domain

VSNKALPAPIEKTISKAKGQPREPQVYTLPPSREEMTKNQVSLTCLVKGFYPSDIAVEWESNGQPENN

IgG1 heavy chain CH3 domain

YKTTPPVLDSDGSFFLYSKLTVDKSRWQQGNVFSCSVMHEALHNHYTQKSLSLSPGK

Figure S2.

The analysis of IFN-β antiproliferative effect inhibited by antibodies produced by hybridomas B1-B21.

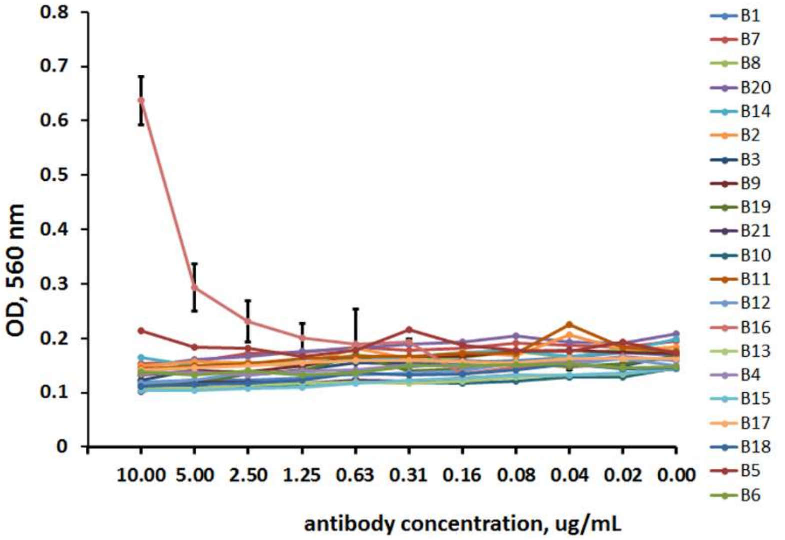

Figure S3. Scheme of sandwich ELISA with simultaneous binding of two antigens: IFN-β and ecdErbB2.

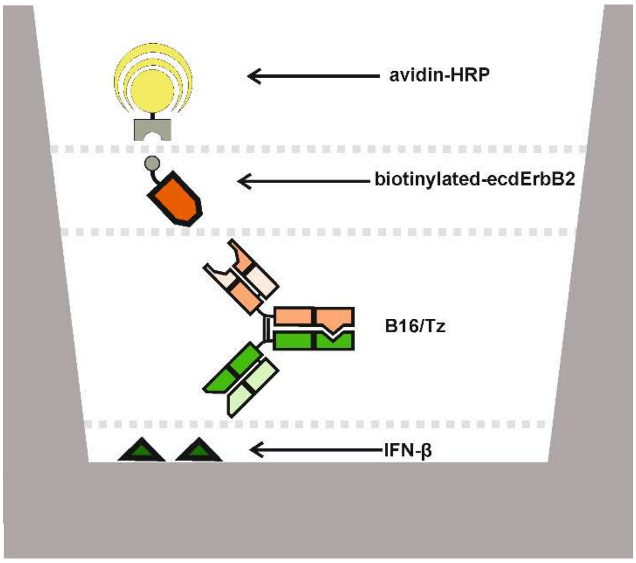

Supplement: Supplementary file 1 [file biomolecules-11-01915-s001.zip › biomolecules-1503781-supplementary.pdf]
